# Biomarkers, Cognitive Function, and Mortality in Centenarians

**DOI:** 10.1001/jamanetworkopen.2026.11335

**Published:** 2026-05-07

**Authors:** Ryo Shikimoto, Takashi Sasaki, Yukiko Abe, Kenji Tai, Nobuyoshi Hirose, Hideyuki Okano, Yasumichi Arai

**Affiliations:** 1 Center for Supercentenarian Medical Research, Keio University School of Medicine, Tokyo, Japan; 2 Department of Neuropsychiatry, Keio University School of Medicine, Tokyo, Japan; 3 Health/Consultation Comprehensive Support Organization, Tokyo University of Agriculture and Technology, Tokyo, Japan; 4 Eisai-Keio Innovation Laboratory for Dementia, Neurology Discovery, DHBL, Eisai Co, Ltd, Tokyo, Japan; 5 Keio University Regenerative Medicine Research Center, Kanagawa, Japan; 6 Faculty of Nursing and Medical care, Keio University School of Medicine, Tokyo, Japan

## Abstract

**Importance:**

Blood-based neural biomarkers linked to aging may provide insights into the biological end point of the human lifespan. However, the key biomarker associated with cognition and mortality in centenarians remains unclear.

**Objective:**

To investigate the associations between 3 neural biomarkers—amyloid-β42 and amyloid-β40 ratio (Aβ42/40), phosphorylated tau 181 (p-tau181), and neurofilament light chain (NfL)—and both cognitive function and all-cause mortality in centenarians.

**Design, Setting, and Participants:**

This population-based cohort study included Japanese centenarians aged 100 years or older who were enrolled between September 2000 and January 2021. Participants underwent baseline cognitive assessments and blood sampling and were followed up for 17 years for mortality. Data analysis was performed in February 2026.

**Exposures:**

Baseline plasma levels of Aβ42/40, p-tau181, and NfL measured using ultrasensitive immunoassays.

**Main Outcomes and Measures:**

Cognitive function at baseline, measured using the Mini-Mental State Examination (MMSE), and all-cause mortality.

**Results:**

Of 495 participants (398 [80.4%] women; mean [SD] age 104.1 [3.0] years), 419 completed a cognitive assessment (mean [SD] MMSE, 14.9 [6.9]). During 17 years of follow-up, 466 participants (95.5%) died. Lower Aβ42/40 (β = 0.99; 95% CI, 0.46 to 1.52) and higher NfL levels (β = −0.92; 95% CI, −1.62 to −0.23) were significantly associated with lower MMSE scores after adjusting for confounders. Higher NfL levels were also associated with increased mortality (hazard ratio, 1.36; 95% CI, 1.17 to 1.57), showing the greatest point estimate among the biomarkers, all of which were standardized and statistically significant (change in Akaike Information Criterion, likelihood ratio test, χ^2^ = 30.16; *P* < .001). Aβ42/40 and p-tau181 were not statistically significant after full adjustment.

**Conclusions and Relevance:**

In this cohort study of centenarians, higher plasma NfL levels were associated with lower cognitive function and increased all-cause mortality, whereas Aβ42/40 and p-tau181 showed no associations. These findings suggest that plasma NfL was associated with neurodegeneration in extreme aging. Further studies are needed to confirm its clinical utility before routine implementation.

## Key Points

**Question:**

Are plasma amyloid-β42 and 40 (Aβ42/40), phosphorylated tau 181 (p-tau181), and neurofilament light chain (NfL) associated with cognitive function and all-cause mortality in centenarians?

**Findings:**

In this cohort study of 495 centenarians aged 100 years or older, lower Aβ42/40 and higher plasma NfL levels were associated with lower cognitive function, higher NfL levels were also associated with increased risk of all-cause mortality. There were no significant associations for Aβ42/40 and p-tau181.

**Meaning:**

These findings suggest plasma NfL may serve as a robust biomarker of neurodegeneration and mortality in extreme aging.

## Teaser

This cohort study evaluates the associations between plasma amyloid-β42 and 40, phosphorylated tau 181, and neurofilament light chain and both cognitive function and all-cause mortality in centenarians in Japan.

## Introduction

Research on age-related neural changes now emphasizes their interactions with broader systemic processes, rather than considering them in isolation.^1,2,3,4,5^ Significant progress in understanding neuronal aging has largely come from studies of neurodegenerative diseases, including the accumulation of misfolded proteins such as amyloid-β and phosphorylated tau.^6^ However, strategies targeting classical protein aggregates like amyloid-β and phosphorylated tau have not fully prevented cognitive decline, often reducing progression by only 20% to 30%, which remains insufficient.^7,8^ Moreover, dementia linked to these pathologies appears less prevalent among the oldest individuals, suggesting alternative neurodegenerative mechanisms may emerge.^9^ Notably, some centenarians preserve cognitive function despite substantial amyloid-β and phosphorylated tau deposition, or maintain executive function especially in socially relevant contexts despite deficits detected by conventional tests.^10^ These observations highlight the need for novel markers to better characterize neural aging in super-aged populations.

Blood biomarkers related to the nervous system, such as amyloid-β, phosphorylated tau181 (p-tau181), and NfL, have been extensively studied for their associations with cognitive function across various diseases and populations.^11,12^ Amyloid-β and p-tau181 are well-established markers for Alzheimer disease onset, severity, and prognosis. They are also associated with all-cause dementia.^13,14^ In contrast, NfL is recognized as a marker of cognitive decline in non-Alzheimer dementias and conditions such as vascular damage, multiple sclerosis, Parkinson disease, and delirium.^15^ Among older adults without dementia, blood phosphorylated tau and NfL have also been reported to be associated with cognitive function.^16,17^ However, no single biomarker has yet been consistently validated in the general population. Furthermore, reports on biomarkers associated with cognitive function in centenarians remain extremely limited. While some studies have linked blood amyloid P and TNF-α to cognitive function,^18,19^ no studies have yet examined correlations between recently established high-precision biomarkers, including NfL and cognitive function. Recently, Nishimoto et al^20^ showed that centenarians exhibit a distinct cognitive function profile. Therefore, research is needed to identify biomarkers associated with cognitive function in populations aged 100 years and older. It is important to interpret these findings specifically among centenarians, as conditioning on age older than 100 could introduce bias.

On the other hand, mortality is associated with various pathological and physiological factors. Serum total phosphorylated tau and NfL have been associated with all-cause mortality in general populations.^21,22,23^ In centenarians, the most reliable factor associated with mortality has been a comprehensive assessment of physical and cognitive function.^24^ We also reported that cardiovascular and inflammatory blood markers such as N-terminal pro–B-type natriuretic peptide (NT-proBNP), interleukin-6, cystatin C, and cholinesterase^25,26^ are associated with mortality in centenarians. Kaeser et al^27^ reported in a study including 135 centenarians that plasma NfL levels in old age were equally or more strongly associated with mortality compared with previously described multi-item scales of cognitive or physical function. These findings highlight the importance of establishing select neurodegenerative markers most strongly associated with mortality in centenarians.

These findings support the idea that a comprehensive assessment of physical and neurological functions, together with relevant biomarkers, is critical for understanding factors associated with mortality risk in aging. However, no study, to our knowledge, has yet examined how blood-based neurodegenerative markers are simultaneously associated with cognition and mortality in the same cohort. In this study, we analyzed NfL levels alongside traditional markers (amyloid-β and p-tau181) and highlighted that NfL levels are associated with both cognitive function and mortality in centenarians.

## Methods

### Study Population

Japanese centenarians were recruited from 2000 to 2021 across 2 prospective cohort studies: the Tokyo Centenarian Study and Japanese Semi-supercentenarian Study.^28,29,30,31^ A total of 1008 participants provided informed consent during the study period. The present study represents a secondary analysis of this long-term cohort. Participants were included in the analytic sample if they had available data for at least 1 neural blood biomarker (amyloid-β40, amyloid-β42, p-tau181, or NfL). Among the 1008 consented participants, 495 met this criterion and were included in the present analysis (Figure). The remaining 513 participants were excluded because none of these biomarker data were available. No other exclusion criteria were applied. The analytic sample consisted of younger centenarians (100-104 years), semi-supercentenarians (105-109 years), and supercentenarians (≥110 years) at enrollment.

**Figure.**
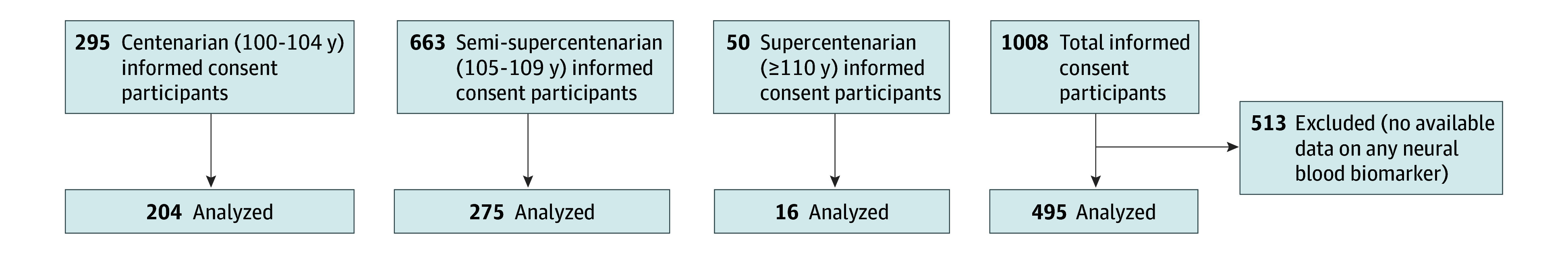
Flowchart Representing the Derivation of the Analytic Sample

The study complied with the Declaration of Helsinki. Written informed consent was obtained from participants or their proxies when participants lacked decision-making capacity. All cohort protocols were approved by the ethics committee of Keio University School of Medicine and registered in the University Hospital Medical Information Network Clinical Trial Registry (ID: UMIN000040446, UMIN000040447, UMIN000001842). This study adhered to the Strengthening the Reporting of Observational Studies in Epidemiology (STROBE) reporting guideline for cohort studies.

### Procedures

Baseline assessment consisted of an in-home or institutional interview, a self-administered questionnaire supported by a primary caregiver, and a clinic-based examination. Sex assigned at birth and age in years were confirmed using official insurance cards issued by the Japanese government. Participants were assessed and examined by trained geriatricians according to protocols described previously.^32,33^

### Measures

#### Neural Blood Biomarkers

Neural blood biomarker measurements were available for all participants included in the present analysis. Due to factors such as biobanking procedures and limited sample availability, the number of samples analyzed for each biomarker varied. Plasma biomarkers were quantified using ultrasensitive immunoassays. Detailed laboratory procedures and quality control information are provided in the eMethods in Supplement 1.

#### Cognitive Function

Cognitive function was assessed using the Mini-Mental State Examination (MMSE).^34^ The Clinical Dementia Rating (CDR) scale^35^ was also administered to evaluate dementia severity. These assessments were conducted by trained psychologists and physicians with expertise in cognitive evaluation.

#### Other Baseline Assessments and Mortality

Demographic characteristics, including body mass index (BMI), education, alcohol use, smoking status, living situation, and medical comorbidities, were evaluated by physician assessment and caregiver-provided information. Basic activities of daily living were assessed using the Barthel Index. Mortality data were obtained from follow-up surveys conducted with participants or their families or caregivers over a period of up to 18 years.

### Statistical Analysis

Plasma biomarkers were log-transformed and standardized to *z* scores. Associations between biomarkers and baseline cognitive function were evaluated using multiple linear regression models. Three models with sequential adjustments for potential confounders were constructed. Covariates were selected based on prior literature and theoretical relevance.^36^ Mortality risk was assessed using Cox proportional hazards models adjusted for demographic and clinical covariates.^37,38^ Nested Cox models were used to evaluate the incremental model fit of biomarkers using changes in Akaike Information Criterion (AIC) and likelihood ratio tests. Missing data were handled using multiple imputation by chained equations. Two-sided tests were used to calculate *P* values, and significance was set at *P* < .05. Detailed statistical procedures are provided in the eMethods in Supplement 1. All analyses were performed in February 2026 using SPSS version 29.0 (IBM).

## Results

### Participant Characteristics

Table 1 shows the characteristics of the participants. Of the 495 participants, 398 (80.4%) were women, and the mean (SD) age was 104.1 (3.0) years. Cognitive function assessments were conducted on 419 individuals, including 76 individuals with missing data who were imputed using multiple imputation, with a mean (SD) MMSE score of 13.7 (7.2). Among the 436 participants with a CDR score, 83 (16.8%) had a score of 0, 59 (11.9%) had a score of 0.5, and 129 (26.1%) had a score of 1. The distributions of plasma biomarkers were as follows: amyloid-β40 ranged from 27.7 to 318.2 pg/mL, amyloid-β42 from 4.0 to 43.5 pg/mL, the amyloid-β42/amyloid-β40 ratio from 0.060 to 0.160, p-tau181 from 11.7 to 162.0 pg/mL, and NfL from 12.5 to 1239.5 pg/mL. Neurofilament light chain levels showed substantial variability (mean [SD], 114.6 [108.4] pg/mL). During up to 17 years of follow-up, 466 participants (95.5%) died (Table 1).

**Table 1.** Characteristics of Participants (N = 495)

Characteristic	Participants, No. (%)	Missing (original)^a^
Baseline information		
Sex		
Female	398 (80.4)	0
Male	97 (19.6)	0
Age, mean (SD), y	104.1 (3.0)	0
Centenarian, 100-104 y	204 (41.2)	0
Semisupercentenarian, 105-109 y	275 (55.6)	0
Supercentenarian, ≥110 y	16 (3.2)	0
ApoE4 positive	44 (8.8)	8
Education, high school or higher	202 (40.7)	14
Alcohol current	76 (15.4)	35
Smoking current	6 (1.2)	6
Facility residents	263 (53.1)	12
Body mass index, mean (SD)^b^	19.0 (5.1)	159
Barthel index score, mean (SD)	47.0 (31.3)	13
Hypertension	329 (66.5)	76
Hyperlipidemia	71 (16.9)	74
Diabetes	42 (8.4)	74
Cerebrovascular disease	111 (26.6)	78
Chronic heart disease	84 (17.0)	78
Stroke	78 (15.7)	78
Cancer	53 (11.1)	18
Surgery	303 (63.3)	16
MMSE, mean (SD)	13.7 (7.2)	76
eGFR, mean (SD)	51.9 (20.6)	0
Amyloid-β40, mean (SD)	116.1 (28.6)	21
Amyloid-β42, mean (SD)	10.9 (3.0)	21
Amyloid-β42/amyloid-β40 ratio, mean (SD)	0.095 (0.015)	21
p-tau181, Mean (SD)	48.4 (20.1)	31
Neurofilament light chain, mean (SD)	114.6 (108.4)	124
Follow-up information		
Follow-up period, mean (SD) [range], d	849.5 (730.4) [15-6223]	9
Death	466 (95.5)	NA
Censored	22 (4.5)	NA

Abbreviations: eGFR, estimated glomerular filtration rate; MMSE, mini-mental state examination; NA, not applicable; p-tau181, phosphorylated tau 181.

^a^ Missing values were imputed using multiple imputation by chained equations, and pooled estimates are reported.

^b^ Calculated as weight in kilograms divided by height in meters squared.

### Association of Plasma Biomarkers With Cognitive Function

Table 2 presents the association of plasma levels of amyloid-β40, amyloid-β42, the amyloid-β42/amyloid-β40 ratio, p-tau181, and NfL with MMSE scores. In model 1, adjusted for sex and age, the Aβ42/Aβ40 (β = 1.27; 95% CI, 0.67 to 1.87), p-tau181 (β = −1.03; 95% CI, −1.64 to −0.42), and NfL (β = −1.83; 95% CI, −2.47 to −1.19) were significantly associated with MMSE scores, with all biomarkers assessed per SD increase. In model 3, which adjusted for potential confounders (biomarkers and other covariates), only the Aβ42/Aβ40 ratio (β = 0.99; 95% CI, 0.46 to 1.52), and NfL (β = −0.92; 95% CI, −1.62 to −0.23) remained significantly associated with MMSE in the final model. The variance inflation factor for all covariates was less than 5 (Table 2).

**Table 2.** Association of Plasma Levels of Amyloid-β40, Amyloid-β42, the Amyloid-β42/Amyloid-β40 Ratio, Phosphorylated Tau 181 (p-tau181), and Neurofilament Light Chain (NfL) With Mini-Mental State Examination Score

Biomarker^d^	Model 1^a^		Model 2^b^		Model 3^c^	
	B (95% CI)	FDR-*P*^e^	B (95% CI)	FDR-P^e^	B (95% CI)	FDR-*P*^e^
Amyloid-β40 (per SD increase)	−1.24 (0.00 to 0.05)	.06	−1.19 (−2.12 to −0.26)	.02	−1.34 (−2.28 to −0.40)	.01
Amyloid-β42 (per SD increase)	0.22 (−0.40 to 0.83)	.49	1.59 (0.71 to 2.47)	<.001	1.70 (0.82 to 2.58)	<.001
Amyloid-β42/amyloid-β40 ratio (per SD increase)	1.27 (0.67 to 1.87)	<.001	0.91 (0.38 to 1.43)	.002	0.99 (0.46 to 1.52)	<.001
p-tau181 (Per SD increase)	−1.03 (−1.64 to −0.42)	.002	−0.37 (−0.96 to 0.23)	.23	−0.31 (−0.90 to 0.29)	.32
NfL (per SD increase)	−1.83 (−2.47 to −1.19)	<.001	−0.92 (−1.60 to −0.24)	.008	−0.92 (−1.62 to −0.23)	.009

Abbreviation: FDR, false discovery rate.

^a^ Model 1 (individual): adjusted for sex, age, and individual biomarkers.

^b^ Model 2 (simultaneous): in addition to sex and age, this model includes adjustments for ApoE4 positivity, education level (high school or higher), Barthel Index score, and biomarkers simultaneously. Model 2 for the Amyloid-β42/amyloid-β40 ratio: in this model, we adjusted for sex, age, ApoE4 positivity, education, Barthel Index score, and plasma levels of p-tau181 and NfL, excluding the individual Amyloid-β40 and Amyloid-β42 values, as these are used to calculate the ratio.

^c^ Model 3 (simultaneous): in addition to the variables in Model 2, this model also adjusts for hypertension, diabetes, chronic heart disease, and stroke.

^d^ Plasma biomarkers were imputed using multiple imputation by chained equations, log-transformed, and standardized as *z* scores.

^e^ *P* values were adjusted for multiple testing (FDR) across biomarkers.

eTable 1 in Supplement 1 shows the results of the sensitivity analysis using complete-case data; sample sizes for models 1 to 3 were 296, 284, and 235, respectively. The direction of point estimates for all biomarkers was consistent with the findings in the full dataset (Table 2), but only NfL in model 1 and the amyloid-β42/amyloid-β40 ratio in model 3 showed significant associations with MMSE scores (eTable 1 in Supplement 1).

### Survival Analysis and Hazard Ratios

Table 3 presents the results of the Cox regression analysis for mortality, adjusted for potential confounders and biomarkers. In model 1, adjusted for sex and age, higher levels of Aβ42/Aβ40 ratio, p-tau181, and NfL were significantly associated with increased mortality. Among these, NfL showed the strongest association (hazard ratio [HR], 1.39; 95% CI, 1.22 to 1.59; *P* < .001). In model 2, which adjusted for additional covariates (ApoE4 positivity, education, Barthel Index score, comorbidities, and estimated glomerular filtration rate [eGFR]), Aβ42/Aβ40 ratio (HR, 1.10; 95% CI, 0.94 to 1.30) and NfL (HR, 1.36; 95% CI, 1.17 to 1.57) remained significantly associated with mortality. p-tau181 was no longer significant (HR, 1.08; 95% CI, 0.95 to 1.22; *P* = .43). Model 3, which adjusted for all biomarkers and covariates, confirmed NfL as most strongly associated with mortality (HR, 1.36; 95% CI, 1.17 to 1.57). The model fit was significantly improved with the addition of biomarkers, as indicated by change in AIC (−28.16; LRT χ^2^ = 30.16; LTL *P* < .001) (Table 3). eTable 2 in Supplement 1 presents the results of the sensitivity analysis for mortality, using complete-case data (231 participants). In model 1, adjusted for sex and age, higher NfL levels were significantly associated with increased mortality (HR, 1.70; 95% CI, 1.42 to 2.05; *P* < .001). This association remained significant in model 2 after adjusting for additional covariates (HR, 1.81; 95% CI, 1.49 to 2.19; *P* < .001). No other biomarkers showed significant associations. These results confirm NfL as most strongly associated with mortality, consistent with the primary analysis (eTable 2 in Supplement 1).

**Table 3.** Cox Regression Analysis for Mortality With Biomarkers (N = 486)

Model type and biomarker^a^^,^^b^	HR (95% CI)	*P* value/FDR-*P*^c^	Change in AIC^d^	LRT χ^2^^d^	LTL *P* value^d^
Model 0 (base)					
Sex (woman)	0.52 (0.40 to 0.66)	<.001	NA	NA	NA
Age	1.06 (1.03 to −1.10)	<.001	NA	NA	NA
ApoE 4 positive	1.00 (0.72 to 1.38)	.98	NA	NA	NA
Education, high school or higher	1.06 (0.87 to 1.31)	.55	NA	NA	NA
Barthel Index Score	0.99 (0.99 to 1.00)	<.001	NA	NA	NA
MMSE	0.98 (0.97 to 1.00)	.04	NA	NA	NA
Hypertension	1.07 (0.86 to 1.32)	.57	NA	NA	NA
Diabetes	1.33 (0.90 to 1.97)	.15	NA	NA	NA
Chronic heart disease	1.05 (0.80 to 1.37)	.72	NA	NA	NA
Stroke	0.86 (0.64 to 1.15)	.31	NA	NA	NA
eGFRcreat	1.00 (0.99 to 1.00)	.09	NA	NA	NA
Model 1 (individual)					
Amyloid-β40	1.12 (0.99 to 1.26)	.10	−1.18	3.18	.08
Amyloid-β42	1.13 (1.01 to 1.25)	.05	−2.85	4.85	.03
Amyloid-β42/amyloid-β40 ratio	1.04 (0.95 to 1.14)	.40	1.24	0.76	.40
p-tau181	1.15 (1.02 to 1.29)	.06	−3.98	5.98	.02
NfL	1.39 (1.22 to 1.59)	<.001	−28.16	30.16	<.001
Model 2 (simultaneous)					
Amyloid-β40	0.94 (0.78 to 1.13)	.51	NA	NA	NA
Amyloid-β42	1.10 (0.94 to 1.30)	.62	NA	NA	NA
p-tau181	1.08 (0.95 to 1.22)	.43	NA	NA	NA
NfL	1.36 (1.17 to 1.57)	<.001	NA	NA	NA
Model 3 (simultaneous)					
Amyloid-β42/amyloid-β40 ratio	1.06 (0.96 to 1.17)	.36	NA	NA	NA

Abbreviations: AIC, Akaike Information Criterion; eGFRcreat, estimated glomerular filtration rate and creatinine; FDR, false discovery rate; LRT, likelihood ratio test; LTL, likelihood test for linearity; MMSE, mini-mental state examination; NA, not applicable; NfL, neurofilament light chain; p-tau181, phosphorylated tau 181.

^a^ All biomarkers were log-transformed and standardized as *z* scores before analysis.

^b^ Model 0 (base): adjusted for sex, age, ApoE 4 positivity, education, Barthel Index, MMSE, physical comorbidities (hypertension, diabetes, heart disease, and stroke), and eGFRcreat. Model 1 (individual): same as Model 0 plus individual biomarkers (amyloid-β40, amyloid-β42, p-tau181, and NfL). Model 2 (simultaneous): same as Model 0 plus all biomarkers (amyloid-β40, amyloid-β42, p-tau181, and NfL). Model 3 (simultaneous): same as Model 0 plus all biomarkers (amyloid-β42/amyloid-β40 ratio, p-tau181, and NfL). Note: The amyloid-β42/amyloid-β40 ratio is derived from 40 and 42, so it is treated separately in model 3 to avoid collinearity.

^c^ *P* value/FDR-P: *P* values for individual biomarkers (amyloid-β40, amyloid-β42, p-tau181, and NfL) were adjusted for multiple comparisons using FDR correction.

^d^ The change in AIC, LRT χ2, and LTL *P* values represent the change in model fit when all biomarkers are added simultaneously to model 0 (base).

### Correlations Between Biomarkers and Clinical Variables

In the supplementary correlation heat map 1, log-transformed NfL was positively correlated with age (*r* = 0.26) and residence status (facility) (*r* = 0.23) and negatively with Hb (*r* = −0.25), albumin (*r* = −0.30), and eGFR (*r* = −0.31), all *P* < .001 after Bonferroni correction (eFigure 1 in Supplement 1). Supplementary correlation heatmap 2 showed associations between blood biomarkers and MMSE scores. Log amyloid-β42 (*r* = −0.18 to −0.13; *P* < .001), log amyloid-β42/amyloid-β40 (*r* = 0.10 to 0.24; *P* < .001), log p-tau181 (*r* = −0.19 to −0.09; *P* < .001), and log NfL (*r* = −0.33 to −0.13; *P* < .001) showed correlations with a broader range of MMSE subscales (eFigure 2 in Supplement 1).

## Discussion

Our study highlights the role of plasma NfL as a key biomarker associated with cognitive function and mortality in centenarians. NfL levels were significantly associated with MMSE scores after adjusting for potential confounders. Moreover, *z* score standardized NfL levels were significantly associated with increased mortality, with each 1 SD increase in NfL corresponding to a 1.36-fold higher risk of death (HR, 1.36; 95% CI, 1.17 to 1.57). These findings position NfL as a guiding marker not only of cognitive decline but also of mortality beyond 100 years, suggesting that a broad range of age-related psychophysiological processes in the brain may influence extreme longevity. It is important to interpret these results specifically among centenarians, as conditioning on age greater than 100 years could introduce bias.

### Factors Associated With Cognitive Function

Our data confirm that decreased Aβ42/Aβ40 ratio and elevated NfL levels are associated with lower cognitive performance, even after controlling for confounders, suggesting that they may reflect underlying neurodegenerative processes. This aligns with previous studies reporting associations between NfL and cognitive function not only in AD^39^ but also in all-cause dementia,^38^ and in various non-AD diseases,^11^ including Parkinson disease, cardiovascular disease, and frontotemporal dementia,^11,15,40^ as well as in being associated with future cognitive decline.^17^ Similarly, decreased Aβ42/Aβ40 ratio has also been linked to cognitive impairment and neurodegenerative diseases. Although previous studies have linked circulating NfL levels to mortality in centenarians,^27^ the association between NfL and cognitive function in this exceptionally long-lived population has not been comprehensively characterized. Centenarians show substantial variability in cognitive function, with some displaying amyloid and phosphorylated tau accumulation without dementia, suggesting that NfL may reflect this neuropathological heterogeneity.^41^ Additionally, neurovascular disorders and non-AD pathologies commonly affect cognition in centenarians, positioning both NfL and Aβ42/Aβ40 ratio as a stronger correlate by capturing a broader range of underlying brain changes. In terms of exposure over time, centenarians likely experience long-term neurodegeneration, and NfL levels may serve as a marker associated with cognitive decline during this process.^42^ Therefore, our results align with the role of NfL in reflecting nonspecific neuronal injury, which may capture a broader spectrum of neurodegeneration, including cerebrovascular pathology and other neurodegenerative conditions prevalent in centenarians.^10,42^ In contrast, p-tau181 showed no association with cognitive function in our cohort. Amyloid-β and tau are believed to play important roles in the onset of Alzheimer disease,^43,44,45^ but there is ongoing debate regarding their relationship and their association with cognitive outcomes. In fact, a meta-analysis of the association between plasma ATN and cognitive function concluded that Amyloid-β was inconsistent, that p-tau181 was strongly associated with cognitive impairment and AD, and that NfL was associated across a wide range of participants. This finding diverges from the established role of these biomarkers in AD pathology,^28,29,38,46,47^ suggesting that centenarians may exhibit different patterns of neurodegenerative processes compared with those populations.^30,31^

### Factors Associated With Mortality

Our longitudinal survival analysis revealed that NfL showed the strongest association with mortality in centenarians, with higher NfL levels corresponding to an increased risk of death. This finding aligns with previous studies indicating that NfL levels have been reported to be associated with mortality in aging populations, including centenarians.^21,23,27,48^ However, the interpretation should be made with caution, as conditioning on age greater than 100 years could introduce bias. Notably, NfL appeared to be more strongly associated with mortality than Aβ or p-tau181, suggesting that nonspecific neuronal injury, as reflected by NfL, may better represent the terminal stages of cognitive aging. Plasma NfL has also been associated with systemic factors such as BMI and cardiovascular disease.^49^ Moreover, as suggested in previous studies, our findings support the association between NfL levels and systemic health markers, including eGFR, albumin, and hemoglobin, all of which are known to influence longevity^46^. This reinforces the idea that NfL serves as a broad indicator of overall health in the aging organism. The lack of association between Aβ42/40 and p-tau181 with mortality in our study is particularly noteworthy. Some previous research has reported that serum total phosphorylated tau is associated with mortality.^22^ However, our study measured p-tau181, and we did not find an association between p-tau181 and mortality in centenarians. While Aβ deposition has long been considered a hallmark of AD, and p-tau181 is a related protein to Aβ, they appear to be less strongly associated with mortality in centenarians compared with neurodegeneration-associated biomarkers such as NfL. This suggests that nonamyloid-β pathology or systemic pathology affecting neurodegeneration rather than amyloid-β pathology itself may play a more significant role in the morbidity and mortality of centenarians, which is compatible with recent findings showing a distinct pattern of cognitive decline in centenarians compared with patients with AD.^20^ The role of physical system decline in chronic inflammation, worsening nutritional status, and declining kidney function as reflected in NfL levels underscores the complex systemic interactions that likely influence mortality in this population.

### Limitations

While our study presents compelling evidence for NfL being strongly associated with cognitive decline and mortality in centenarians, certain limitations warrant consideration. First, this study did not account for a formal diagnosis of dementia. Since participants with a CDR of 0 to 0.5 represented only a quarter of the total, the number of participants without dementia was insufficient for meaningful statistical analysis. Second, reliance on blood-based biomarkers limits direct comparison with brain-specific pathology, such as neurofibrillary tangles and cerebrovascular lesions. However, recent advancements in blood-based biomarker technologies are promising, and in our study, we employed highly sensitive assays that have demonstrated validity through correlation with brain PET imaging,^47^ offering a noninvasive method to examine neuropathological processes in centenarians.^12^ Third, although the large sample size and long follow-up period strengthen our findings, the study cohort was limited to a Japanese population. Replication in other ethnic groups and regions will be essential to evaluate the generalizability of these findings and to examine potential cultural or genetic influences on NfL as a biomarker of aging. Additionally, as our study was observational and cross-sectional in nature, causal inferences cannot be definitively established.

## Conclusions

In this cohort study of centenarians, we found that NfL was a promising biomarker associated with cognitive decline and mortality. Unlike Aβ42/40 and p-tau181, which were associated with AD, NfL appeared to be a more generalizable marker of neurodegeneration that reflected the complex interactions between the nervous system and physiological systems such as immunity and vascular function. These findings suggest that NfL could serve as a valuable biomarker for assessing the health trajectory of extremely old adults and provide critical insights into the mechanisms underlying cognitive aging at the limits of human life. Further research, including longitudinal analyses, neuroimaging, and autopsy-based studies, is required to elucidate the role of NfL in the aging brain and to explore its potential as a biomarker for extending healthy lifespan.

## Notes

Supplement 1. eMethods. eTable 1. Sensitivity Analysis Using Complete-Case Data: Association of Plasma Levels of Amyloid-β40, Amyloid-β42, the Amyloid-β42/Amyloid-β40 Ratio, p-tau181, and NfL With MMSE Score eTable 2. Sensitivity Analysis Using Complete-Case Data: Cox Regression Analysis for Survival With Biomarkers eFigure 1. Heatmap of Associations Between Blood Biomarkers and Clinical Variables eFigure 2. Heatmap of Associations Between MMSE Scores and Blood Biomarkers

Supplement 2. Data Sharing Statement

## References

[1] Johansen MC, Ye W, Gross A, . Association between acute myocardial infarction and cognition. JAMA Neurol. 2023;80(7):723-731. doi:10.1001/jamaneurol.2023.1331 37252710 PMC10230369

[2] Tang X, Han YP, Chai YH, . Association of kidney function and brain health: a systematic review and meta-analysis of cohort studies. Ageing Res Rev. 2022;82:101762. doi:10.1016/j.arr.2022.101762 36374833

[3] Tesi N, van der Lee SJ, Hulsman M, . Immune response and endocytosis pathways are associated with the resilience against Alzheimer’s disease. Transl Psychiatry. 2020;10(1):332. doi:10.1038/s41398-020-01018-7 32994401 PMC7524800

[4] Shaulson ED, Cohen AA, Picard M. The brain-body energy conservation model of aging. Nat Aging. 2024;4(10):1354-1371. doi:10.1038/s43587-024-00716-x 39379694

[5] López-Otín C, Blasco MA, Partridge L, Serrano M, Kroemer G. The hallmarks of aging. Cell. 2013;153(6):1194-1217. doi:10.1016/j.cell.2013.05.039 23746838 PMC3836174

[6] Aman Y, Schmauck-Medina T, Hansen M, . Autophagy in healthy aging and disease. Nat Aging. 2021;1(8):634-650. doi:10.1038/s43587-021-00098-4 34901876 PMC8659158

[7] Sims JR, Zimmer JA, Evans CD, ; TRAILBLAZER-ALZ 2 Investigators. Donanemab in early symptomatic alzheimer disease: the TRAILBLAZER-ALZ 2 randomized clinical trial. JAMA. 2023;330(6):512-527. doi:10.1001/jama.2023.13239 37459141 PMC10352931

[8] van Dyck CH, Swanson CJ, Aisen P, . Lecanemab in early Alzheimer’s disease. N Engl J Med. 2023;388(1):9-21. doi:10.1056/NEJMoa2212948 36449413

[9] Nelson PT, Trojanowski JQ, Abner EL, . “New old pathologies”: AD, PART, and cerebral age-related TDP-43 with sclerosis (CARTS). J Neuropathol Exp Neurol. 2016;75(6):482-498. doi:10.1093/jnen/nlw033 27209644 PMC6366658

[10] Zhang M, Ganz AB, Rohde S, . The correlation between neuropathology levels and cognitive performance in centenarians. Alzheimers Dement. 2023;19(11):5036-5047. doi:10.1002/alz.13087 37092333

[11] Garcia-Escobar G, Manero RM, Fernández-Lebrero A, . Blood biomarkers of Alzheimer’s disease and cognition: a literature review. Biomolecules. 2024;14(1):93. doi:10.3390/biom14010093 38254693 PMC10813472

[12] Olsson B, Lautner R, Andreasson U, . CSF and blood biomarkers for the diagnosis of Alzheimer’s disease: a systematic review and meta-analysis. Lancet Neurol. 2016;15(7):673-684. doi:10.1016/S1474-4422(16)00070-3 27068280

[13] Mattsson-Carlgren N, Salvadó G, Ashton NJ, . Prediction of longitudinal cognitive decline in preclinical Alzheimer disease using plasma biomarkers. JAMA Neurol. 2023;80(4):360-369. doi:10.1001/jamaneurol.2022.5272 36745413 PMC10087054

[14] Karikari TK, Pascoal TA, Ashton NJ, . Blood phosphorylated tau 181 as a biomarker for Alzheimer’s disease: a diagnostic performance and prediction modelling study using data from four prospective cohorts. Lancet Neurol. 2020;19(5):422-433. doi:10.1016/S1474-4422(20)30071-5 32333900

[15] Fohner AE, Bartz TM, Tracy RP, . Association of serum neurofilament light chain concentration and MRI findings in older adults: the cardiovascular health study. Neurology. 2022;98(9):e903-e911. doi:10.1212/WNL.0000000000013229 34921102 PMC8901174

[16] McGrath ER, Beiser AS, O’Donnell A, . Blood phosphorylated tau 181 as a biomarker for amyloid burden on brain PET in cognitively healthy adults. J Alzheimers Dis. 2022;87(4):1517-1526. doi:10.3233/JAD-215639 35491781

[17] Kern S, Syrjanen JA, Blennow K, . Association of cerebrospinal fluid neurofilament light protein with risk of mild cognitive impairment among individuals without cognitive impairment. JAMA Neurol. 2019;76(2):187-193. doi:10.1001/jamaneurol.2018.3459 30419087 PMC6439969

[18] Nybo M, Olsen H, Jeune B, Andersen-Ranberg K, Holm Nielsen E, Svehag SE. Increased plasma concentration of serum amyloid P component in centenarians with impaired cognitive performance. Dement Geriatr Cogn Disord. 1998;9(3):126-129. doi:10.1159/000017035 9621998

[19] Bruunsgaard H, Andersen-Ranberg K, Jeune B, Pedersen AN, Skinhøj P, Pedersen BK. A high plasma concentration of TNF-alpha is associated with dementia in centenarians. J Gerontol A Biol Sci Med Sci. 1999;54(7):M357-M364. doi:10.1093/gerona/54.7.M357 10462168

[20] Nishimoto Y, Sasaki T, Abe Y, . Distinct patterns of cognitive traits in extreme old age and Alzheimer’s disease. Alzheimers Dement. 2025;21(4):e70155. doi:10.1002/alz.70155 40235113 PMC12000243

[21] Régy M, Dugravot A, Sabia S, . Association between ATN profiles and mortality in a clinical cohort of patients with cognitive disorders. Alzheimers Res Ther. 2023;15(1):77. doi:10.1186/s13195-023-01220-x 37038213 PMC10088112

[22] Halloway S, Evans DA, Desai P, Dhana K, Beck T, Rajan KB. Serum total tau, neurofilament light, and glial fibrillary acidic protein are associated with mortality in a population study. J Am Geriatr Soc. 2024;72(1):149-159. doi:10.1111/jgs.18632 37818793 PMC10842309

[23] Rübsamen N, Maceski A, Leppert D, . Serum neurofilament light and tau as prognostic markers for all-cause mortality in the elderly general population-an analysis from the MEMO study. BMC Med. 2021;19(1):38. doi:10.1186/s12916-021-01915-8 33583409 PMC7883435

[24] Mossakowska M, Broczek K, Wieczorowska-Tobis K, . Cognitive performance and functional status are the major factors predicting survival of centenarians in Poland. J Gerontol A Biol Sci Med Sci. 2014;69(10):1269-1275. doi:10.1093/gerona/glu003 24509978

[25] Szewieczek J, Francuz T, Dulawa J, . Functional measures, inflammatory markers and endothelin-1 as predictors of 360-day survival in centenarians. Age (Dordr). 2015;37(5):85. doi:10.1007/s11357-015-9822-9 26289439 PMC5005827

[26] Hirata T, Arai Y, Yuasa S, . Associations of cardiovascular biomarkers and plasma albumin with exceptional survival to the highest ages. Nat Commun. 2020;11(1):3820. doi:10.1038/s41467-020-17636-0 32732919 PMC7393489

[27] Kaeser SA, Lehallier B, Thinggaard M, . A neuronal blood marker is associated with mortality in old age. Nat Aging. 2021;1(2):218-225. doi:10.1038/s43587-021-00028-4 37118632

[28] Gondo Y, Hirose N, Arai Y, . Functional status of centenarians in Tokyo, Japan: developing better phenotypes of exceptional longevity. J Gerontol A Biol Sci Med Sci. 2006;61(3):305-310. doi:10.1093/gerona/61.3.305 16567382

[29] Arai Y, Iinuma T, Takayama M, . The Tokyo Oldest Old survey on Total Health (TOOTH): a longitudinal cohort study of multidimensional components of health and well-being. BMC Geriatr. 2010;10:35. doi:10.1186/1471-2318-10-35 20529368 PMC2893189

[30] Arai Y, Inagaki H, Takayama M, . Physical independence and mortality at the extreme limit of life span: supercentenarians study in Japan. J Gerontol A Biol Sci Med Sci. 2014;69(4):486-494. doi:10.1093/gerona/glt146 24225329

[31] Arai Y, Martin-Ruiz CM, Takayama M, . Inflammation, but not telomere length, predicts successful ageing at extreme old age: a longitudinal study of semi-supercentenarians. EBioMedicine. 2015;2(10):1549-1558. doi:10.1016/j.ebiom.2015.07.029 26629551 PMC4634197

[32] Preische O, Schultz SA, Apel A, ; Dominantly Inherited Alzheimer Network. Serum neurofilament dynamics predicts neurodegeneration and clinical progression in presymptomatic Alzheimer’s disease. Nat Med. 2019;25(2):277-283. doi:10.1038/s41591-018-0304-3 30664784 PMC6367005

[33] de Wolf F, Ghanbari M, Licher S, . Plasma tau, neurofilament light chain and amyloid-β levels and risk of dementia; a population-based cohort study. Brain. 2020;143(4):1220-1232. doi:10.1093/brain/awaa054 32206776 PMC7174054

[34] Beker N, Ganz A, Hulsman M, . Association of cognitive function trajectories in centenarians with postmortem neuropathology, physical health, and other risk factors for cognitive decline. JAMA Netw Open. 2021;4(1):e2031654. doi:10.1001/jamanetworkopen.2020.31654 33449094 PMC7811180

[35] Koyama A, Okereke OI, Yang T, Blacker D, Selkoe DJ, Grodstein F. Plasma amyloid-β as a predictor of dementia and cognitive decline: a systematic review and meta-analysis. Arch Neurol. 2012;69(7):824-831. doi:10.1001/archneurol.2011.1841 22451159 PMC3772635

[36] van Oijen M, Hofman A, Soares HD, Koudstaal PJ, Breteler MMB. Plasma Abeta(1-40) and Abeta(1-42) and the risk of dementia: a prospective case-cohort study. Lancet Neurol. 2006;5(8):655-660. doi:10.1016/S1474-4422(06)70501-4 16857570

[37] Lim YY, Maruff P, Kaneko N, . Plasma amyloid-β biomarker associated with cognitive decline in preclinical Alzheimer’s disease. J Alzheimers Dis. 2020;77(3):1057-1065. doi:10.3233/JAD-200475 32925048

[38] Li Z, Fan Z, Zhang Q. The associations of phosphorylated tau 181 and tau 231 levels in plasma and cerebrospinal fluid with cognitive function in Alzheimer’s disease: a systematic review and meta-analysis. J Alzheimers Dis. 2024;98(1):13-32. doi:10.3233/JAD-230799 38339929

[39] Nakamura A, Kaneko N, Villemagne VL, . High performance plasma amyloid-β biomarkers for Alzheimer’s disease. Nature. 2018;554(7691):249-254. doi:10.1038/nature25456 29420472

[40] Zabala-Findlay A, Penny LK, Lofthouse RA, . Utility of blood-based tau biomarkers for mild cognitive impairment and Alzheimer’s disease: systematic review and meta-analysis. Cells. 2023;12(8):1184. doi:10.3390/cells12081184 37190093 PMC10136726

[41] Chen YR, Liang CS, Chu H, . Diagnostic accuracy of blood biomarkers for Alzheimer’s disease and amnestic mild cognitive impairment: a meta-analysis. Ageing Res Rev. 2021;71:101446. doi:10.1016/j.arr.2021.101446 34391944

[42] Davey A, Dai T, Woodard JL, ; Georgia Centenarian. Profiles of cognitive functioning in a population-based sample of centenarians using factor mixture analysis. Exp Aging Res. 2013;39(2):125-144. doi:10.1080/0361073X.2013.761869 23421635 PMC3579538

[43] Andersen SL. Centenarians as models of resistance and resilience to Alzheimer’s disease and related dementias. Adv Geriatr Med Res. 2020;2(3):e200018. doi:10.20900/agmr20200018 32743561 PMC7394313

[44] Beydoun MA, Noren Hooten N, Weiss J, . Plasma neurofilament light and its association with all-cause mortality risk among urban middle-aged men and women. BMC Med. 2022;20(1):218. doi:10.1186/s12916-022-02425-x 35692046 PMC9190073

[45] Bavato F, Barro C, Schnider LK, . Introducing neurofilament light chain measure in psychiatry: current evidence, opportunities, and pitfalls. Mol Psychiatry. 2024;29(8):2543-2559. doi:10.1038/s41380-024-02524-6 38503931 PMC11412913

[46] Sarto J, Esteller-Gauxax D, Tort-Merino A, . Impact of demographics and comorbid conditions on plasma biomarkers concentrations and their diagnostic accuracy in a memory clinic cohort. J Neurol. 2024;271(4):1973-1984. doi:10.1007/s00415-023-12153-8 38151575

[47] Mattsson N, Andreasson U, Zetterberg H, Blennow K; Alzheimer’s Disease Neuroimaging Initiative. Association of plasma neurofilament light with neurodegeneration in patients with Alzheimer disease. JAMA Neurol. 2017;74(5):557-566. doi:10.1001/jamaneurol.2016.6117 28346578 PMC5822204

[48] Folstein MF, Folstein SE, McHugh PR. “Mini-mental state”. a practical method for grading the cognitive state of patients for the clinician. J Psychiatr Res. 1975;12(3):189-198. doi:10.1016/0022-3956(75)90026-6 1202204

[49] Morris JC. Clinical dementia rating: a reliable and valid diagnostic and staging measure for dementia of the Alzheimer type. Int Psychogeriatr. 1997;9(suppl 1):173-176. doi:10.1017/S1041610297004870 9447441

