# Supplementary material for: Biomarkers, Cognitive Function, and Mortality in Centenarians
Source: JAMA Netw Open. 2026 May 7;9(5):e2611335. doi: 10.1001/jamanetworkopen.2026.11335 (PMC13153989; doi:10.1001/jamanetworkopen.2026.11335)
Supplement: Supplement 1. — eMethods. eTable 1. Sensitivity Analysis Using Complete-Case Data: Association of Plasma Levels of Amyloid-β40, Amyloid-β42, the Amyloid-β42/Amyloid-β40 Ratio, p-tau181, and NfL With MMSE Score eTable 2. Sensitivity Analysis Using Complete-Case Data: Cox Regression Analysis for Survival With Biomarkers eFigure 1. Heatmap of Associations Between Blood Biomarkers and Clinical Variables eFigure 2. Heatmap of Associations Between MMSE Scores and Blood Biomarkers [file jamanetwopen-e2611335-s001.pdf]

Supplemental Online Content

Shikimoto R, Sasaki T, Abe Y, et al. Neurofilament light chain, cognitive function, and mortality in centenarians. *JAMA Netw Open*. 2026;9(5):e2611335. doi:10.1001/jamanetworkopen.2026.11335

eMethods.
eTable 1. Sensitivity Analysis Using Complete-Case Data: Association of Plasma Levels of Amyloid-β40, Amyloid-β42, the Amyloid-β42/Amyloid-β40 Ratio, p-tau181, and NfL With MMSE Score
eTable 2. Sensitivity Analysis Using Complete-Case Data: Cox Regression Analysis for Survival With Biomarkers
eFigure 1. Heatmap of Associations Between Blood Biomarkers and Clinical Variables
eFigure 2. Heatmap of Associations Between MMSE Scores and Blood Biomarkers

This supplemental material has been provided by the authors to give readers additional information about their work.

© 2026 Shikimoto R et al. *JAMA Network Open*.

eMethods.

Study population

Japanese centenarians were recruited from 2000 to 2021 across two prospective cohort studies: the Tokyo Centenarian Study (TCS) (20), and Japanese Semi-supercentenarian Study (JSS) [30,31]. A total of 1,008 participants provided informed consent during the study period. The present study represents a secondary analysis of this long-term cohort. Participants were included in the analytic sample if they had available data for at least one neural blood biomarker (amyloid-β40, amyloid-β42, p-tau181, or NfL). Among the 1,008 consented participants, 495 met this criterion and were included in the present analysis. The remaining 513 participants were excluded because none of these biomarker data were available. No other exclusion criteria were applied. The analytic sample consisted of 495 centenarians, including 204 younger centenarians (100–104 years), 275 semi-supercentenarians (105–109 years), and 16 supercentenarians (≥110 years) at enrollment.

We complied with all relevant ethical regulations for research involving human participants, including the tenets of the Declaration of Helsinki. Written informed consent was obtained from either the participants or their proxies if the individuals lacked the capacity to consent. All cohort protocols were approved by the ethics committee of Keio University School of Medicine and registered as observational studies in the University Hospital Medical Information Network Clinical Trial Registry (ID: UMIN000040446, UMIN000040447, UMIN000001842). This study adheres to the STROBE reporting guideline for cohort studies.

Procedures

Baseline assessment consisted of an in-home or institutional interview, a self-administered questionnaire supported by a primary caregiver, and a clinic-based examination. Sex assigned at

© 2026 Shikimoto R et al. *JAMA Network Open*.

birth and age were confirmed using official insurance cards issued by the Japanese government. Participants were assessed and examined by trained geriatricians according to protocols described previously[28,29].

Measures

Neural Blood Biomarkers

Neural blood biomarker measurements were available for 495 participants included in the present analysis. Due to factors such as biobanking procedures and limited sample availability, the number of samples analyzed for each biomarker varied: amyloid β 42/40 (Aβ 42/40) (n=474), phosphorylated tau 181 (p-tau181) (n=464), and Neurofilament Light (NfL) (n=371). Plasma biomarkers were measured as follows: Aβ 42/40 was quantified using the Human/Rat β Amyloid (40) ELISA Kit Wako II and the Human/Rat β Amyloid (42) ELISA Kit Wako. p-tau181 and NfL were assayed using Simoa (Single Molecule Array) technology, with the High-Sensitive Simoa™ p-tau181 Advantage Kit v2.1 (104111) and the Simoa NF-Light Advantage Kit v2 (104073), respectively. Analyses were conducted using the Simoa HD-1 Analyzer™ and the Simoa HD-X Analyzer™. Additionally, we measured comprehensive blood biomarkers for physical assessment: the genetic variant ApoE4, complete blood count, comprehensive metabolic panel, and inflammatory biomarkers. ApoE4 is a known risk factor for AD and is also recognized as a genetic variant associated with longevity. The NfL and p-tau181 levels were measured using the Simoa NF-Light Advantage Kit v2 on both HD-1 and HD-X Simoa analyzers. Quality control (QC) assessments were performed for each run, with results within the specified range. The lower limit of quantification (LLOQ) for p-tau181 was 1.91 pg/mL, and the upper limit of quantification (ULOQ) was 470 pg/mL. The coefficient of variation (CV%) for

each run ranged from 0.56% to 11.82% based on the QC standards (QC-STD 1, 2). All measurements were within the LLOQ-ULOQ range.

Cognitive function

Neuropsychologists screened mental health and assessed cognition using the Mini-Mental State Examination (MMSE)[32] and the CDR scale[33]. The MMSE and CDR were administered by trained professionals, including psychologists and physicians, with expertise in cognitive assessments. Objective evaluation of the CDR was based on information provided by family members or facility caregivers.

Other physical and psychological evaluations at baseline

Demographics, including BMI, education, alcohol use, smoking status, living situation (home or facility), and comorbidities (such as hypertension, hyperlipidemia, diabetes, chronic heart disease, respiratory diseases, renal diseases, cerebrovascular diseases, dementia, fractures, and prior surgeries), were evaluated by physicians through participant observation and caregiver-provided information. The comorbidity list was consistent with those used in the TCS and JSS studies and was classified according to ICD-10. Basic activities of daily living were assessed using the Barthel Index. The quality, coherence, and completeness of the data were reviewed by an experienced geriatrician during the examination.

Overall mortality

Mortality data were obtained from follow-up surveys conducted with participants or their families/caregivers over a period of up to 18 years.

Statistics

Cross-sectional analysis

© 2026 Shikimoto R et al. *JAMA Network Open.*

Descriptive statistics, including frequencies (N, %) for categorical variables and mean ± SD, range for continuous variables, were used to summarize baseline demographics and health measures. Plasma biomarkers were log-transformed and standardized to Z-scores. Associations between plasma levels of amyloid-β40, amyloid-β42, the amyloid-β42/amyloid-β40 ratio, p-tau181, and neurofilament light (NfL) with MMSE scores were evaluated using multiple linear regression models. Model 1 (Individual) was adjusted for sex, age, and individual biomarkers. Model 2 (Simultaneous) included additional adjustments for sex, age, ApoE4 positivity, education level (high school or higher), Barthel Index score, and biomarkers (amyloid-β40, amyloid-β42, p-tau181, and NfL) simultaneously. For the amyloid-β42/amyloid-β40 ratio, Model 2 was modified to include only p-tau181 and NfL, while retaining the other covariates (sex, age, ApoE4 positivity, education level, Barthel Index score), excluding the individual amyloid-β40 and amyloid-β42 values, as these are used to calculate the ratio. Finally, Model 3 (Simultaneous) included additional adjustments for hypertension, diabetes, chronic heart disease, and stroke. These covariates included sex, age, ApoE4 positivity, education level (high school or higher), Barthel Index score, past history of hypertension, diabetes, chronic heart disease, and stroke. These variables were selected based on their established relevance in prior literature and their theoretical significance in relation to cognitive function and mortality[34] .

Longitudinal Analysis

To investigate associations between cognitive function, as assessed by MMSE total score, biomarkers, and mortality, survival analysis was conducted using Cox regression models. Plasma biomarkers were log-transformed, and standardized as Z-scores. Cox regression models were adjusted for sex, age, ApoE4 positivity, education level (high school or higher), Barthel Index Score, MMSE, physical comorbidities (hypertension, diabetes, heart disease, stroke), and

© 2026 Shikimoto R et al. *JAMA Network Open.*

eGFRcreat, and log-transformed and standardized biomarkers. These variables were selected based on their established relevance in prior literature and their theoretical significance in relation to cognitive function and mortality, as well as following the analytical approaches commonly used in studies involving biomarkers, where eGFR is often included as an important physiological marker[35,36]. Nested Cox models were used to assess the added value of each biomarker, with ΔAIC and likelihood-ratio tests quantifying improvement in model fit. The ΔAIC, LRT χ^2, and LTL p-value values represent the change in model fit when all biomarkers are added simultaneously to Model 0 (Base). P-values for individual biomarkers (Amyloid-β40, Amyloid-β42, p-tau181, and NfL) were adjusted for multiple comparisons using False Discovery Rate (FDR) correction to control for the increased risk of type I errors due to multiple testing. All analyses were performed using SPSS v29.0 (IBM Corp., Chicago, IL, USA).

Missing data imputation

Missing data were handled using multiple imputation by chained equations (MICE) for plasma biomarkers (log-transformed amyloid β40, amyloid β42, p-tau181, neurofilament light, and ApoE4 positivity), individual MMSE items, and covariates with missing values, including education level, Barthel Index, comorbidities (hypertension, diabetes, chronic heart disease, and stroke), and eGFR. All covariates used in the regression and survival models were included in the imputation model. Sex and age had no missing values and were included as covariates but not imputed. The MMSE total score was calculated from the imputed individual items. The Clinical Dementia Rating (CDR) and mortality outcomes (time to death or censoring) were not imputed due to the ordinal or time-to-event nature of these variables.

The FCS model used 20 imputations and 20 iterations, with constraints to preserve valid ranges for MMSE items and a fixed seed (1234567) for reproducibility. Convergence was confirmed by

© 2026 Shikimoto R et al. *JAMA Network Open.*

examining the mean and standard deviation of imputed values across iterations using SPSS Imputation Summary tables; values were stable. Analyses used pooled estimates across imputations according to Rubin's rules. For descriptive statistics, pooled standard deviations (SD) were calculated as the square root of the mean of the variances across the 20 imputed datasets.

Data availability

The analyzed datasets are restricted due to the inclusion of sensitive information and are available upon request with approval from the Keio University School of Medicine Research Ethics Committee, via the corresponding author.

© 2026 Shikimoto R et al. *JAMA Network Open*.

eTable 1a. Sensitivity Analysis Using Complete-Case Data: Association of Plasma Levels of Amyloid-β40, Amyloid-β42, the Amyloid-β42/Amyloid-β40 Ratio, p-tau181, and NfL With MMSE Score (N=296).

	Model 1				Model 2				Model 3			
	B (95%CI)			FDR-P	B (95%CI)			FDR-P	B (95%CI)			FDR-P
Amyloid-β40 (per SD increase)	-0.44	(-1.21	- 0.32)	.42	-0.76	(-2.01	- 0.49)	.29	-1.31	(-2.63	- 0.02)	.09
Amyloid-β42 (per SD increase)	0.02	(-0.73	- 0.76)	.97	1.14	(-0.02	- 2.30)	.28	1.499	(0.30	- 2.70)	.07
Amyloid-β42/amyloid-β40 ratio (per SD increase)	0.69	(-0.05	- 1.42)	.17	0.62	(-0.05	- 1.29)	.17	**0.866**	**(0.17**	**- 1.56)**	**.04**
p-tau181 (per SD increase)	-0.39	(-1.14	- 0.37)	.39	-0.25	(-1.02	- 0.51)	.51	-0.08	(-0.92	- 0.76)	.85
NfL (per SD increase)	**-1.42**	**(-2.15**	**-- 0.68)**	**<.001**	-0.61	(-1.44	- 0.21)	.24	-0.608	(-1.51	- 0.29)	.23

This table presents the associations between plasma biomarkers (amyloid-β40, amyloid-β42, amyloid-β42/amyloid-β40 ratio, p-tau181, and NfL) and Mini-Mental State Examination (MMSE) scores. The values are presented as regression coefficients (B) with 95% confidence intervals (CI) and p-values. Statistical adjustments were made for various confounders (sex, age, ApoE4 positivity, education, Barthel Index Score, etc.). The ranges of coefficients and p-values are provided for each model.

Cases with missing values were excluded; sample sizes for Models 1–3 were 296, 284, and 235, respectively.

Plasma biomarkers were imputed using MICE, log-transformed, and standardized as Z-scores.

P-values were adjusted for multiple testing (FDR) across three biomarkers.

Model 1: Adjusted for sex, age.

Model 2: + additional adjustment for ApoE4 positive, education (high school or higher), Barthel Index Score, and plasma levels of other biomarkers.

Model 2 for amyloid-β42/amyloid-β40 ratio: Model 2 + additional adjustment only for plasma levels of p-tau181 and NfL.

Model 3: + additional adjustment for Hypertension, Diabetes, Chronic Heart Disease, and Stroke.

NfL, neurofilament light chain

*FDR-P < .05 is shown in bold

eTable 2. Sensitivity Analysis Using Complete-Case Data: Cox Regression Analysis for Survival With Biomarkers (N=231)

	Model type	HR (95%CI)			P-value/FDR-p	ΔAIC	LRT χ^2	LTL p-value
**sex (woman)**	Model 0 (Base)	**0.47**	**0.33**	**0.67**	**<.001**			
**age**	Model 0 (Base)	**1.07**	**1.01**	**1.14**	**.02**			
ApoE 4+	Model 0 (Base)	1.04	0.61	1.76	.89			
Education, high school or higher	Model 0 (Base)	0.97	0.73	1.28	.82			
**Barthel Index Score**	Model 0 (Base)	**0.99**	**0.98**	**1.00**	**<.001**			
**MMSE**	Model 0 (Base)	**0.99**	**0.97**	**1.02**	**.50**			
Hypertension	Model 0 (Base)	1.06	0.78	1.45	.71			
Diabetes	Model 0 (Base)	2.60	1.52	4.46	<.001			
Chronic Heart Disease	Model 0 (Base)	1.13	0.76	1.67	.54			
Stroke	Model 0 (Base)	0.89	0.59	1.35	.57			
eGFRcreat	Model 0 (Base)	1.00	0.99	1.00	.19			
Amyloid-β40	Model 1 (individual)	1.04	0.89	1.22	>.99	1.78	0.22	.64
	Model 2 (Simultaneous)	0.93	0.73	1.18	.90			
**Amyloid-β42**	Model 1 (individual)	1.03	0.90	1.19	>.99	1.79	0.22	.64
	Model 2 (Simultaneous)	0.95	0.76	1.19	.83			
Amyloid-β42/amyloid-β40 ratio	Model 1 (individual)	1.01	0.89	1.14	.91	1.99	0.01	.91
	Model 3 (Simultaneous)	0.99	0.87	1.12	.81			
**p-tau181**	Model 1 (individual)	1.09	0.91	1.30	.92	1.19	0.81	.37
	Model 2 (Simultaneous)	0.94	0.79	1.13	>.99			
**NFL**	**Model 1 (individual)**	**1.70**	**1.42**	**2.05**	**<.001**	**28.17**	**30.17**	**<.001**
	**Model 2 (Simultaneous)**	**1.81**	**1.49**	**2.19**	**<.001**			

Model 0 (Base): Adjusted for sex, age, ApoE 4+, education, Barthel Index, MMSE, physical comorbidities (hypertension, diabetes, heart disease, stroke), and eGFRcreat.
Model 1 (Individual): Same as Model 0 + individual biomarkers (amyloid-β40, amyloid-β42, p-tau181, NFL)
Model 2 (Simultaneous): Same as Model 0 + all biomarkers (amyloid-β40, amyloid-β42, p-tau181, NFL).
Model 3 (Simultaneous): Same as Model 0 + all biomarkers (amyloid-β42/amyloid-β40 ratio, p-tau181, NFL).
Note: The amyloid-β42/amyloid-β40 ratio is derived from 40 and 42, so it is treated separately in Model 3 to avoid collinearity.
All biomarkers were log-transformed and standardized as Z-scores before analysis.
The ΔAIC, LRT χ^2, and LTL p-value values represent the change in model fit when all biomarkers are added simultaneously to Model 0 (Base).
P-value/FDR-p: P-values for individual biomarkers (amyloid-β40, amyloid-β42, p-tau181, and NFL) were adjusted for multiple comparisons using False Discovery Rate (FDR) correction.
*P/FDR-p < .05 is shown in bold

© 2026 Shikimoto R et al. *JAMA Network Open.*

eFigure 1. Heatmap of Associations Between Blood Biomarkers and Clinical Variables

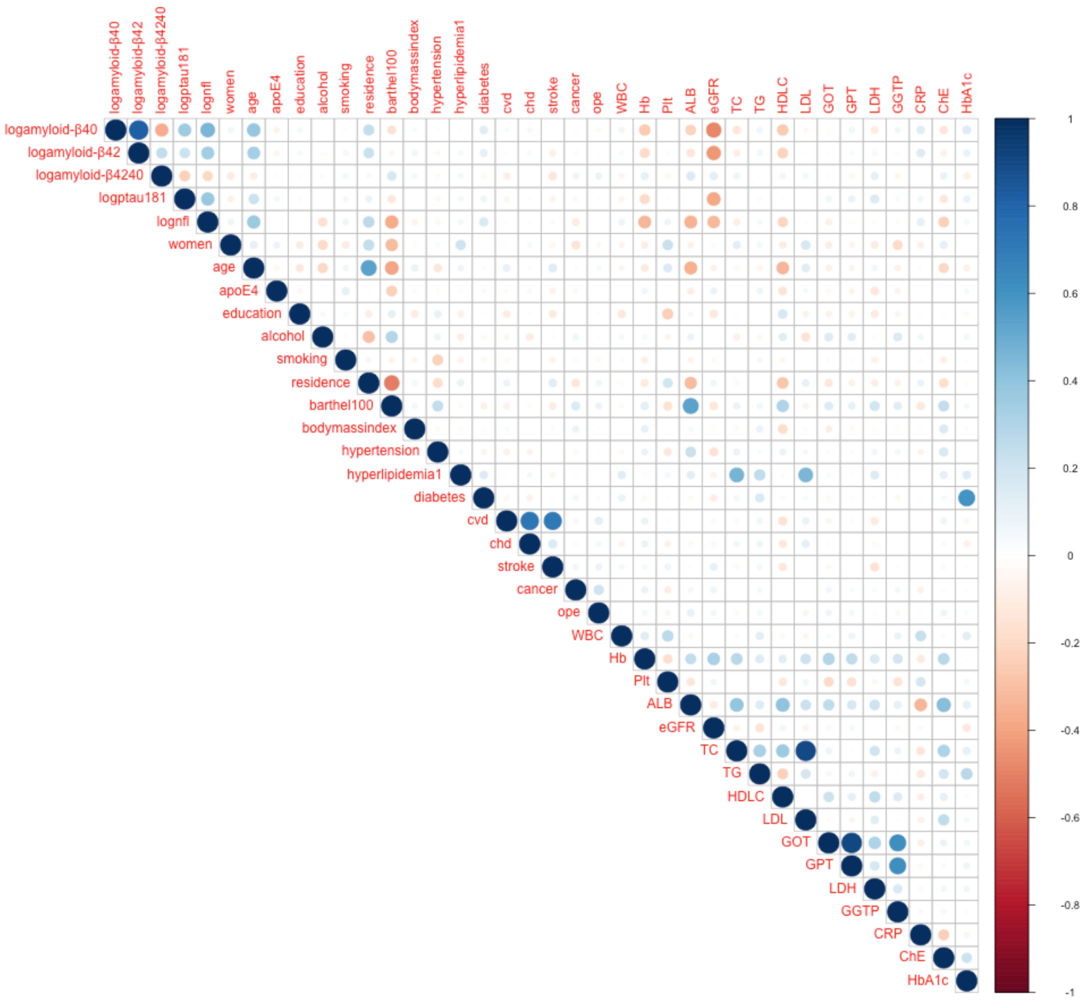

The relationships between selected clinical and biochemical variables were assessed using pairwise correlation analysis.

A heterogeneous correlation approach was applied to account for differing data types (e.g., continuous, ordinal, and categorical variables).

Correlation coefficients, ranging from −1 to +1, represent the strength and direction of linear or monotonic relationships.

© 2026 Shikimoto R et al. *JAMA Network Open.*

Positive values indicate positive associations, negative values indicate inverse associations, and values near zero suggest weak or no association.

The heatmap displays the correlation coefficients, with color intensity reflecting the strength of association between variable pairs.

logamyloid-β40, log-transformed amyloid-β40; logamyloid-β42, log-transformed amyloid-β42; logamyloid-β4042, log-transformed amyloid-β42/amyloid-β40 ratio; logptau181, log-transformed phosphorylated tau 181; lognfl, log-transformed neurofilament light chain; cvd, Cerebro Vascular Disease; chd, Chronic Heart Disease; WBC, white blood cells; Hb, hemoglobin; Plt, platelet; ALB, albumin; eGFR, estimated glomerular filtration rate; TC, total cholesterol; TG, triglyceride; HDLC, high-density lipoprotein cholesterol; LDL, low-density lipoprotein cholesterol; GOT, glutamate oxaloacetate transaminase; GPT, glutamate pyruvate transaminase; LDH, lactate dehydrogenase; GGTP, γ-glutamyl transpeptidase; CRP, C-reactive protein; ChE, cholinesterase, HbA1c, hemoglobin A1c.

© 2026 Shikimoto R et al. *JAMA Network Open.*

eFigure 2. Heatmap of Associations Between MMSE Scores and Blood Biomarkers

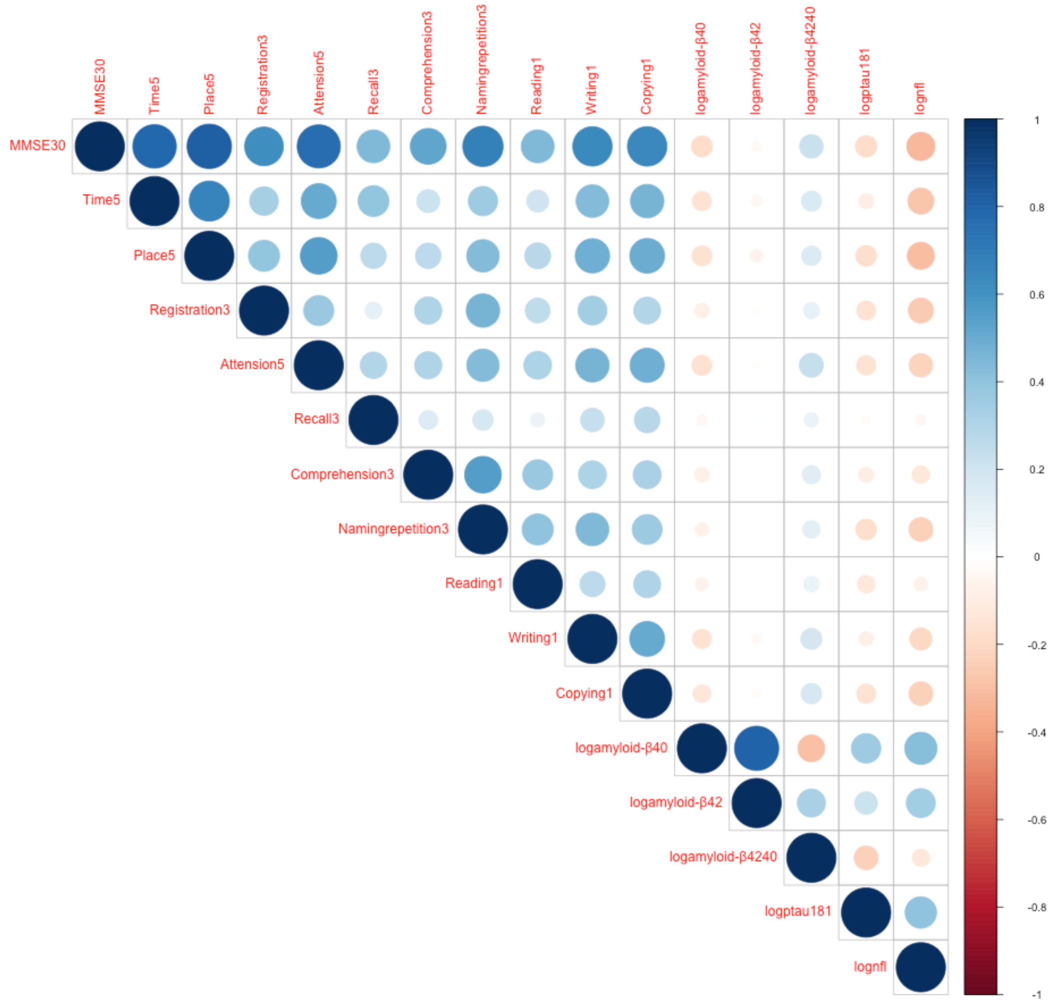

The relationships between selected clinical and biochemical variables were assessed using pairwise correlation analysis.

A heterogeneous correlation approach was applied to account for differing data types (e.g., continuous, ordinal, and categorical variables).

© 2026 Shikimoto R et al. *JAMA Network Open.*

Correlation coefficients, ranging from −1 to +1, represent the strength and direction of linear or monotonic relationships. Positive values indicate positive associations, negative values indicate inverse associations, and values near zero suggest weak or no association.

The heatmap displays these correlation coefficients, with color intensity reflecting the strength of association between variable pairs.

Time5, Time orientation (5-point scale); Place5, Place orientation (5-point scale); Registration3, memory encoding (3-point scale); Attention5, attention and calculation (5-point scale); Recall3, memory retrieval (3-point scale); Comprehension3, language comprehension (3-point scale); Namingrepetition3, naming and repetition(3-point scale); Reading1, reading ability(1-point scale); Writing, writing ability(1-point scale); copying, copying ability(1-point scale); logamyloid-β40, log-transformed amyloid-β40; logamyloid-β42, log-transformed amyloid-β42; logamyloid-β4042, log-transformed amyloid-β42/amyloid-β40 ratio; logptau181, log-transformed phosphorylated tau 181; lognfl, log-transformed Neurofilament Light Chain.

© 2026 Shikimoto R et al. *JAMA Network Open.*
