# Supplementary material for: Biomarkers, Cognitive Function, and Mortality in Centenarians
Source: JAMA Netw Open. 2026 May 7;9(5):e2611335. doi: 10.1001/jamanetworkopen.2026.11335 (PMC13153989; doi:10.1001/jamanetworkopen.2026.11335)
Supplement: Supplement 2. — Data Sharing Statement [file jamanetwopen-e2611335-s002.pdf]

Data Sharing Statement

Shikimoto. Neurofilament Light Chain, Cognitive Function, and Mortality in Centenarians. *JAMA Netw Open*. Published May 07, 2026. doi:10.1001/jamanetworkopen.2026.11335

Data
Data available: No

Additional Information
Explanation for why data not available: The analyzed datasets are restricted due to the inclusion of sensitive information and are available upon request with approval from the Keio University School of Medicine Research Ethics Committee, via the corresponding author.
